# Association Between Ideal Cardiovascular Health and Vegetarian Dietary Patterns Among Community-Dwelling Individuals

**DOI:** 10.3389/fnut.2022.761982

**Published:** 2022-03-18

**Authors:** Yu-Min He, Wei-Liang Chen, Tung-Wei Kao, Li-Wei Wu, Hui-Fang Yang, Tao-Chun Peng

**Affiliations:** ^1^Division of Family Medicine, Department of Family and Community Medicine, National Defense Medical Center, Tri-Service General Hospital, Taipei, Taiwan; ^2^Division of Geriatric Medicine, Department of Family and Community Medicine, National Defense Medical Center, Tri-Service General Hospital, Taipei, Taiwan; ^3^Graduate Institute of Medical Sciences, National Defense Medical Center, Taipei, Taiwan; ^4^Graduate Institute of Clinical Medicine, College of Medicine, National Taiwan University, Taipei, Taiwan

**Keywords:** American Heart Association (AHA), cardiovascular health (CVH), Life's Simple 7 (LS7), vegetarian, cardiovascular disease, risk factor

## Abstract

**Background:**

Vegetarians have been shown to have better metabolic profiles than non-vegetarians, and vegetarianism has potential beneficial effects on cardiovascular disease. However, there is a lack of studies on vegetarians that examine both metabolic profiles and lifestyle habits, such as physical activity, smoking habits, and dietary patterns, which are equally important in the context of cardiovascular disease. We explored whether a vegetarian diet is associated with both metabolic traits and lifestyle habits by assessing cardiovascular health (CVH) metrics.

**Methods:**

This was a cross-sectional study conducted in a Taiwanese population. Data collected between 2000 and 2016 were extracted from the MJ Health database. Participants aged 40 years and older without cardiovascular disease were included. CVH metrics included smoking habits, blood pressure, total cholesterol, serum glucose, body mass index, physical activity, and healthy diet score. Vegetarian participants were full-time vegetarians who did not consume meat or fish. All the data were assessed from self-report questionnaires, physical examinations, and blood analyses following standard protocol. Multiple logistic regression analysis was used to evaluate the association between vegetarianism and CVH metrics.

**Results:**

Of 46,287 eligible participants, 1,896 (4.1%) were vegetarian. Overall, vegetarians had better CVH metrics (OR = 2.09, 95% CI = 1.84–2.37) but lower healthy diet scores (OR = 0.41, 95% CI = 0.33–0.51) after adjustment. No difference in physical activity (OR = 0.86, 95% CI = 0.73–1.02) was identified between vegetarians and non-vegetarians. Additionally, vegetarians had higher whole grain intake (OR = 2.76, 95% CI = 2.28–3.35) and lower sugar-sweetened beverage consumption (OR = 1.36, 95% CI = 1.18–1.58).

**Conclusions:**

Our results suggested that vegetarians had better overall ideal CVH metrics but lower ideal healthy diet scores than non-vegetarians, which was likely due to the lack of fish consumption in this population group. When assessing CVH metrics and healthy diet scores for vegetarians, metrics and scores chosen should be suitable for use with vegetarian populations.

## Introduction

Although the mortality rate from cardiovascular disease (CVD) has dramatically decreased since the 1960s, it is still the leading cause of mortality in the US and worldwide (1, 2). In 2010, with the aim of decreasing the incidence of CVD and improving cardiovascular health, the American Heart Association (AHA) presented the ideal cardiovascular health metric, which is composed of 7 CVD risk factors or behaviors, including smoking habit, blood pressure, total cholesterol, serum glucose, body mass index (BMI), physical activity, and diet (3). People with at least 5 of 7 ideal cardiovascular health (CVH) metrics had a lower risk for heart-related death (4) and diabetes (5). However, the extent to which these metrics differ between non-vegetarian and vegetarian patients in clinical practice is unclear, although it is of particular interest because diet is one of the 7 important components. In addition, dietary patterns have effects on many different risk factors for CVD, such as hypertension, hyperglycemia and hyperlipidemia.

Plant-based and vegetarian diets appear to protect against the risk of CVD (6, 7). Vegetarians commonly report lower rates of metabolic syndrome and cardiometabolic risk factors, such as elevated fasting blood sugar, low-density lipoprotein (LDL), BMI, and waist circumference (8–10). In addition, the adoption of a vegetarian diet has been shown to reduce the risk factors for and mortality rate of CVD in Western populations (11–13). Considering the different dietary habits and populations, several cross-sectional studies performed in Taiwan highlighted similar results (14–16). Vegetarians had a lower risk of obesity, elevated blood pressure, elevated glucose, and diabetes than non-vegetarians in the Taiwanese population (14, 15). These previous studies on vegetarians were mainly focused on direct and measurable cardiovascular risk factors, such as serum glucose, lipoprotein, body weight, and blood pressure (8, 17, 18). Few studies have examined other lifestyle risk factors among vegetarians that are equally important for CVD risk, such as smoking, diet, and physical activity (19, 20). The ideal cardiovascular health metric comprehensively combines cardiometabolic risk factors and lifestyle habits (3). For this reason, the metric is more comprehensive and may play an important role in evaluating the association between vegetarian diets and CVD.

To further investigate this association, our main objective was to assess whether vegetarians have more favorable CVH metrics than non-vegetarians in the Taiwanese population. In addition, the relationship between vegetarian dietary patterns and the healthy diet score CVH metric was examined.

## Methods

### Study Population and Design

**Figure 1:**
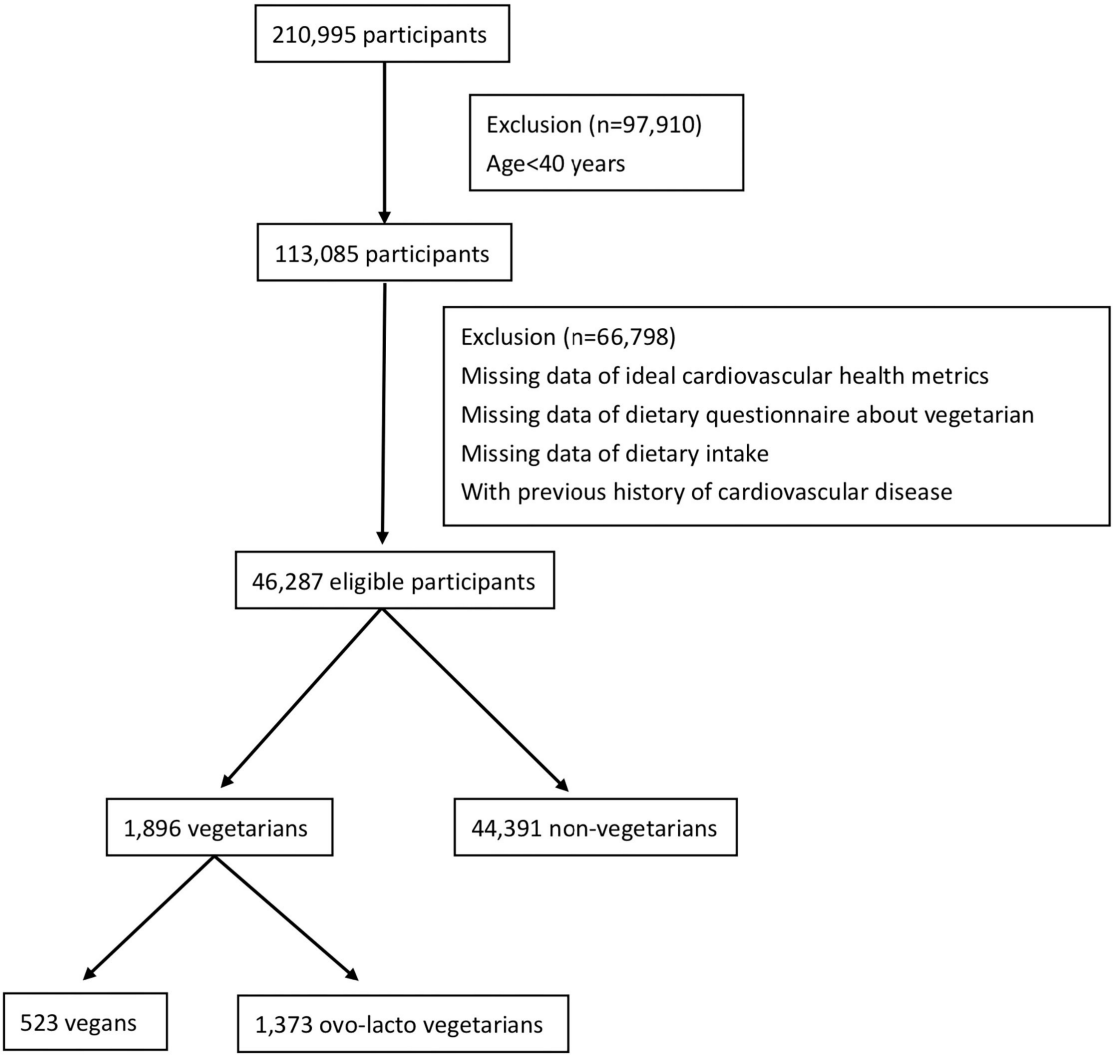
Flow chart of selection of study participants.

This was a cross-sectional study conducted in a Taiwanese population. The data analyzed in our study were extracted from the MJ Health database between 2000 and 2016. The MJ Health database is a large, comprehensive, population-based health database created by MJ Health Management (21). All examinations and equipment from MJ Health Management were certified by the International Organization for Standardization (ISO). The institution has 4 centers distributed across Taiwan and provides health examinations, including questionnaires, physical examinations, and blood analyses. All the protocol and test procedures were the same at all centers. Because the personality and health behaviors were relatively unchanged after middle adulthood age (22, 23), participants aged 40 years and older who underwent MJ health examinations and without any missing CVH metric data between 2000 and 2016 were included. To evaluate the concept of primary prevention, participants with cardiovascular disease were excluded. The flow chart of the selection of study participants is shown in Figure 1. The data were more complete for subjects aged 40 years and above. Among 210,995 subjects screened, 97,910 subjects were excluded due to age under 40 years. Of the remaining participants, 66,798 subjects without ideal CVH metric data or dietary questionnaires about vegetarianism or with a previous history of CVD were excluded. Finally, 46,287 eligible participants were included in our study. The distribution of the participants by year is shown in Supplementary Table S1. All eligible participants completed health questionnaires (including age, sex, education, family income, smoking status, dietary pattern, physical activity, lifestyle, and medical history questionnaires) and underwent physical examinations (including height, body weight, and blood pressure) and blood analysis (including fasting glucose and total cholesterol). All of the data on the questionnaire were self-reported by the participants. The procedures for physical examination and blood analysis were identical and performed with instruments of the same model. Blood samples were collected and analyzed by the same protocol (21). All participants signed a form indicating that they agreed to allow their anonymous personal data to be used for research purposes. The Institutional Review Board of the Tri-Service General Hospital, Taiwan, approved this study.

### Sociodemographic Data

Education was classified into two groups: below or equivalent to high school and beyond high school. Family income, based on all members of household, was calculated as annual income and expressed as New Taiwan dollars (NTDs). A cutoff point of 1.2 million NTD, which was average Taiwanese family income in 2016, was used to divide the participants into two groups.

### Dietary Data

Dietary patterns were assessed through the certified and standardized semiquantitative food frequency questionnaire (FFQ) created by MJ Health Management (24). Before the data collection, the participants needed to answer 85 closed-ended questions about food consumption at different meal times during the month. According to the answers and hypothesized health effects, these would be classified into 22 non-overlapping food questions about intake of different food items or groups according to the characteristics of Taiwanese dietary patterns. The amount was assessed by the intake frequency data, and information on portion size was estimated with the pictures of measuring tools in each question. For example, the description of sugar-sweetened beverage consumption was “How much sugar-sweetened beverage do you drink, including coffee, tea, and Coke? (1 cup is equivalent to 240 mL)”. Each question had 5 choices for intake frequency, including “none or <1 cup a week, 1–3 cups a week, 4–6 cups a week, 1 cup a day, or ≥2 cups a day,” from lowest to highest.

The vegetarians in our study were defined as people without meat or fish consumption and had to meet 2 criteria. First, participants answered yes to the question asking whether they were a full-time vegetarian (always consuming a vegetarian diet). Second, participants answered no fish or other meat consumption in the individual dietary questionnaire. Additionally, we divided the vegetarians into 2 subgroups: vegans and ovo-lacto-vegetarians. Participants who were vegetarian and consumed eggs or milk 1 time/week or more were classified as ovo-lacto vegetarians. Vegetarians who did not consume eggs or milk were classified as vegans. Another vegetarian subgroup was pesco-vegetarians, which means that the subjects consumed fish and vegetables. Due to the different dietary patterns, the population of pesco-vegetarians was small in Taiwan, and data were difficult to collect. Therefore, this subgroup was not analyzed in our study.

### Cardiovascular Health Metrics

Based on the AHA guidelines, the cardiovascular health metric was composed of smoking habit, blood pressure, total cholesterol, serum glucose, BMI, physical activity, and diet. Ratings of ideal, intermediate, and poor were assigned for each component (3).

Smoking status, measured byw a self-reported questionnaire, was categorized as ideal for never smokers, intermediate for past smokers, and poor for current smokers. Blood pressure was measured twice on the right arm with the subject in a sitting position after 5 min of rest. It was classified as ideal, intermediate, and poor for systolic blood pressure (SBP) <120 mmHg and diastolic blood pressure (DBP) <80 mmHg, SBP 120–139 mmHg or DBP 80–89 mmHg or treated to goal, and SBP ≥140 mmHg or DBP ≥90 mmHg, respectively. The term “treated to goal” used in the intermediate health status means that individuals achieve the ideal health status through medication. For cholesterol and fasting glucose, the participant had to fast for more than 8 hours before the blood sample was taken, and the sample was analyzed according to standard protocol (21). Total cholesterol was classified as ideal for <200 mg/dL, intermediate for 200–239 mg/dL or treated to goal, and poor for ≥240 mg/dL. Fasting glucose was categorized as ideal for <100 mg/dL, intermediate for 100–125 mg/dL, and poor for ≥126 mg/dL. BMI was calculated according to height and body weight. Based on the Taiwanese Health Promotion Administration, a healthy BMI of adults in Taiwan is recommended to be between 18.5–24 kg/m^2^, which is different from the AHA guidelines. For comparison to other studies about ideal CVH, we used the definition of the AHA guideline to analyze BMI in our study. It was classified as ideal at <25 kg/m^2^, intermediate at 25–29.9 kg/m^2^, and poor at values ≥30 kg/m^2^. Physical activity was measured by self-reported physical activity questionnaires from MJ Management (25). This questionnaire was similar to International Physical Activity Questionnaire (26), and could record the activity intensity, frequency, and duration to analyze the physical activity of CVH metrics. The ideal, intermediate, and poor values were defined as ≥210 min/week, 60–210 min/week, and <60 min/week of physical activity, respectively. The healthy diet score, obtained from a self-reported dietary questionnaire, was composed of five healthy dietary components, including fruits and vegetables (≥450 g/d), fish (≥198 g/wk), fiber-rich whole grains (≥85 g/d), sodium (<1,500 mg/d), and sugar-sweetened beverages ( ≤1 liter/wk). The ideal, intermediate, and poor healthy diet scores were defined as 4–5 components, 2–3 components, and 0–1 components, respectively (3). Each component of the CVH metric that met the ideal status was assigned one point. A higher score indicated better cardiovascular health.

### Statistical Analysis

The characteristics of our study participants are described as the mean and standard deviation for continuous variables and frequency for categorical variables. Continuous variables, including age, BMI, blood pressure, total cholesterol, and fasting glucose, were evaluated using Student's *t*-test. Categorical variables, including vegetarian status, education level, family income, sex, ideal blood pressure, ideal total cholesterol, ideal fasting glucose, ideal BMI, ideal smoking, ideal physical activity, ideal healthy diet score, and ideal CVH metrics, were analyzed by the chi-square test based on the baseline characteristics. Vegetarian diet was an independent variable, and the CVH metrics were dependent variables. We investigated each outcome separately in our study. Multiple logistic regression analysis was used to assess the association between vegetarian diet status and CVH metrics. Because consumption egg or milk may impact the CVH based on previous studies (27, 28), we divided vegetarians into vegans and ovo-lacto-vegetarians, and performed the subgroup analysis by multiple logistic regression analysis. In addition, the association between vegetarian diet status and different healthy dietary components was also assessed by multiple logistic regression analysis. Non-vegetarian was the reference group. Consumption of eggs or milk was not a component of the healthy diet score. Hence, we suggested that there was no significant difference between the healthy diet scores of each vegetarian subgroup and compared only the vegetarian and non-vegetarian groups. We adjusted for multiple potential confounding factors, including age, sex, education level, and family income. The confounders were referenced from previous literature (29, 30). The analyses were two-tailed, with *P* < 0.05 considered significant, and the data were analyzed with the Statistical Package for the Social Sciences version 22 (SPSS, Inc., Chicago, IL, USA).

## Results

### Demographics

**Figure 2:**
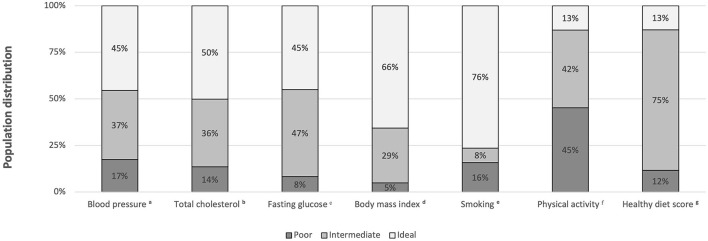
Distribution of each component of the CVH metric in the total analyzed population (vegetarians plus non-vegetarians). BP, blood pressure; SBP, systolic blood pressure; DBP, diastolic blood pressure; BMI, body mass index. ^a^Ideal, intermediate, poor BP were defined as SBP < 120 mmHg and DBP < 80 mmHg, SBP 120-139 mmHg or DBP 80-89 mmHg or treated to goal, and SBP ≥ 140 mmHg or DBP ≥ 90 mmHg, respectively. ^b^Ideal, intermediate, poor total cholesterol were defined as <200 mg/dL, 200–239 mg/dL or treated to goal, and 240 mg/dL, respectively. ^c^Ideal, intermediate, poor fasting glucose were defined as <100 mg/dL, 100–125 mg/dL, and ≥126 mg/dL, respectively. ^d^Ideal, intermediate, poor body mass index (BMI) were defined as <25 kg/m^2^, 25–29.9 kg/m^2^, and ≥30 kg/m^2^, respectively. ^e^Ideal, intermediate, poor smoking status were defined as never smokers, past smokers, and current smokers, respectively. ^f^Ideal, intermediate, and poor physical activity were defined as ≥210 min/week, 60-210 min/week, and < 60 min/week, respectively. ^g^Ideal, intermediate, and poor healthy diet scores were defined as 4–5 components, 2–3 components, and 0–1 components, respectively.

A total of 1,896 (4.1%) vegetarians and 44,391 (95.9%) non-vegetarians were included in our study. Among the vegetarians, 523 (27.6%) subjects were vegans and 1,373 (72.4%) subjects were ovo-lacto vegetarians. Table 1 shows the characteristics of the vegetarians and non-vegetarians. Supplementary Table S2 shows the characteristics of vegan, ovo-lacto vegetarian, and non-vegetarian. Figure 2 shows the distribution of each component of the CVH metric in the analyzed population. Compared to the non-vegetarian group, the vegetarian group had significantly lower BMI (*P* < 0.01), total cholesterol (*P* < 0.01), fasting glucose (*P* < 0.01), and diastolic blood pressure (*P* < 0.01). The vegetarian group had higher proportions of older and female patients (*P* < 0.01). Moreover, vegetarians showed a lower education level (*P* < 0.01) and family income (*P* < 0.01). There was no difference in mean systolic blood pressure (*P* = 0.28).

**Table 1:** Characteristics of the study participants.

**Characteristics**	**All (*n* = 46,287)**	**Vegetarian (*n* = 1,896)**	**Non-vegetarian (*n* = 44,391)**	***P*-value**
**Continuous variables^a^**				
Age, years	52.6 (10.1)	56.1 (10.8)	52.5 (10.0)	<0.01
BMI, kg/m^2^	24.0 (3.4)	23.1 (3.3)	24.0 (3.4)	<0.01
Total cholesterol, mg/dL	201.7 (35.6)	181.7 (33.5)	202.5 (35.4)	<0.01
Fasting sugar, mg/dL	105.8 (24.3)	102.9 (22.2)	106 (24.4)	<0.01
Systolic pressure, mmHg	121.4 (18.8)	121.9 (20.0)	121.4 (18.7)	0.28
Diastolic pressure, mmHg	74.5 (11.6)	73.7 (11.7)	74.5 (11.5)	<0.01
**Categorical variables^b^**				
**Education**				<0.01
Below high school	20,866 (46.8)	1,029 (57.0)	19,837 (46.4)	
beyond high school	23,681 (53.2)	777 (43.0)	22,904 (53.6)	
**Family income, NTD/year^c^**				<0.01
<1.2 million	24,857 (68.2)	1,116 (76.9)	23,741 (67.9)	
>1.2 million	11,571 (31.8)	336 (23.1)	11,235 (32.1)	
**Sex**				<0.01
Male	22,987 (49.7)	639 (33.7)	22,348 (50.3)	
Female	23,300 (50.3)	1,257 (66.3)	22,043 (49.7)	
**Blood pressure^d^**				<0.01
Poor	8,084 (17.5)	352 (18.6)	7,732 (17.4)	
Intermediate	17,147 (37.0)	622 (32.8)	16,525 (37.2)	
Ideal	21,056 (45.5)	922 (48.6)	20,134 (45.4)	
**Total cholesterol^e^**				<0.01
Poor	6,265 (13.5)	96 (5.1)	6,169 (13.9)	
Intermediate	16,841 (36.4)	432 (22.8)	16,409 (37.0)	
Ideal	23,181 (50.1)	1,368 (72.1)	21,813 (49.1)	
**Fasting glucose^f^**				<0.01
Poor	3,859 (8.3)	126 (6.6)	3,733 (8.4)	
Intermediate	21,576 (46.6)	715 (37.7)	20,861 (47.0)	
Ideal	20,852 (45.0)	1,055 (55.6)	19,797 (44.6)	
**Body mass index^g^**				<0.01
Poor	2,262 (4.9)	68 (3.6)	2,194 (5.0)	
Intermediate	13,652 (29.5)	425 (22.4)	13,227 (29.8)	
Ideal	30,373 (65.6)	1,403 (74.0)	28,970 (65.3)	
**Smoking^h^**				<0.01
Poor	7,343 (15.9)	44 (2.3)	7,299 (16.4)	
Intermediate	3,573 (7.7)	105 (5.5)	3,468 (7.8)	
Ideal	35,371 (76.4)	1,747 (92.1)	33,624 (75.7)	
**Physical activity^i^**				0.2
Poor	20,940 (45.2)	887 (46.8)	20,053 (45.2)	
Intermediate	19,283 (41.7)	752 (39.7)	18,531 (41.7)	
Ideal	6,064 (13.1)	257 (13.6)	5,807 (13.1)	
**Healthy diet score^j^**				<0.01
Poor	5,416 (11.7)	153 (8.1)	5,263 (11.9)	
Intermediate	34,905 (75.4)	1,638 (86.4)	33,267 (74.9)	
Ideal	5,966 (12.9)	105 (5.5)	5,861 (13.2)	
**Number of ideal CVH metrics**				<0.01
0–2	15,850 (34.2)	379 (20.0)	15,471 (34.9)	
3–4	23,094 (49.9)	1,014 (53.5)	22,080 (49.7)	
5–7	7,343 (15.9)	503 (26.5)	6,840 (15.4)	

a *Values in the continuous variables were expressed as mean (standard deviation) and analyzed by Student's t-test*.

b *Values in the categorical variables were expressed as number (%) and analyzed by the chi-square test*.

c *Values were expressed as New Taiwan dollar (NTD)*.

d *Ideal, intermediate, poor BP were defined as SBP <120 mmHg and DBP <80 mmHg, SBP 120–139 mmHg or DBP 80–89 mmHg or treated to goal, and SBP ≥140 mmHg or DBP ≥90 mmHg, respectively*.

e *Ideal, intermediate, poor total cholesterol were defined as <200 mg/dL, 200–239 mg/dL or treated to goal, and 240 mg/dL, respectively*.

f *Ideal, intermediate, poor fasting glucose were defined as <100 mg/dL, 100–125 mg/dL, and ≥126 mg/dL, respectively*.

g *Ideal, intermediate, poor BMI were defined as <25 kg/m^2^, 25–29.9 kg/m^2^, and ≥30 kg/m^2^ respectively*.

h *Ideal, intermediate, poor smoking status were defined as never smokers, past smokers, and current smokers, respectively*.

i *Ideal, intermediate, and poor physical activity were defined as ≥210 min/week, 60–210 min/week, and <60 min/week, respectively*.

j *Ideal, intermediate, and poor healthy diet scores were defined as 4–5 components, 2–3 components, and 0–1 components, respectively*.

### Vegetarian Diet and Cardiovascular Health Metric

The relationship between ideal cardiovascular health and vegetarian diet status is shown in Table 2. Compared to non-vegetarians, vegetarians had better blood pressure (OR = 1.14, 95% CI = 1.04–1.25), total cholesterol (OR = 2.68, 95% CI = 2.42–2.97), fasting glucose (OR = 1.56, 95% CI = 1.42–1.71), and BMI (OR = 1.52, 95% CI = 1.37–1.68). Regarding health behaviors, the vegetarian group had a higher non-smoking rate (OR = 3.75, 95% CI = 3.17–4.44). There was no significant difference in physical activity (OR = 1.04, 95% CI = 0.91–1.19). Although vegetarians were more likely to have a lower healthy diet score (OR = 0.39, 95% CI = 0.32–0.47), the prevalence of at least 5 ideal cardiovascular health metrics was higher in the vegetarian group (OR = 1.98, 95% CI = 1.78–2.2).

**Table 2:** The odds ratio for the number of ideal cardiovascular health metrics between vegetarian and non-vegetarian.

	**Odds ratio (95% CI)^a^**	
	**Unadjusted**	**Adjusted^b^**
Blood pressure	1.14 (1.04–1.25)	1.34 (1.20–1.50)
Total cholesterol	2.68 (2.42–2.97)	2.79 (2.48–3.15)
Fasting glucose	1.56 (1.42–1.71)	1.63 (1.46–1.82)
Body mass index	1.52 (1.37–1.68)	1.46 (1.29–1.66)
Smoking	3.75 (3.17–4.44)	2.78 (2.27–3.41)
Physical activity	1.04 (0.91–1.19)	0.86 (0.73–1.02)
Healthy diet score	0.39 (0.32–0.47)	0.41 (0.33–0.51)
Number of ideal CVH metrics^c^	1.98 (1.78–2.20)	2.09 (1.84–2.37)

a *Odds ratio and 95% CI were estimated by logistic regression models and using non-vegetarian as the reference group*.

b *Adjusted for age, gender, education level, and family income*.

c *Number of ideal CVH metrics: 5–7*.

After multivariable adjustment, the vegetarian diets showed a consistent effect on the ideal cardiovascular health metric (OR = 2.09, 95% CI = 1.84–2.37). The vegetarian group was more likely to have normal blood pressure (OR = 1.34, 95% CI = 1.2–1.5), total cholesterol (OR = 2.79, 95% CI = 2.48–3.15), fasting glucose (OR = 1.63, 95% CI = 1.46–1.82), and BMI (OR = 1.46, 95% CI = 1.29–1.66) and exhibited a higher non-smoking ratio (OR = 2.78, 95% CI = 2.27–3.41), whereas a lower healthy diet score was seen in the vegetarian group (OR = 0.41, 95% CI = 0.33–0.51). No significant difference was seen in physical activity between the groups (OR = 0.86, 95% CI = 0.73–1.02).

**Figure 3:**
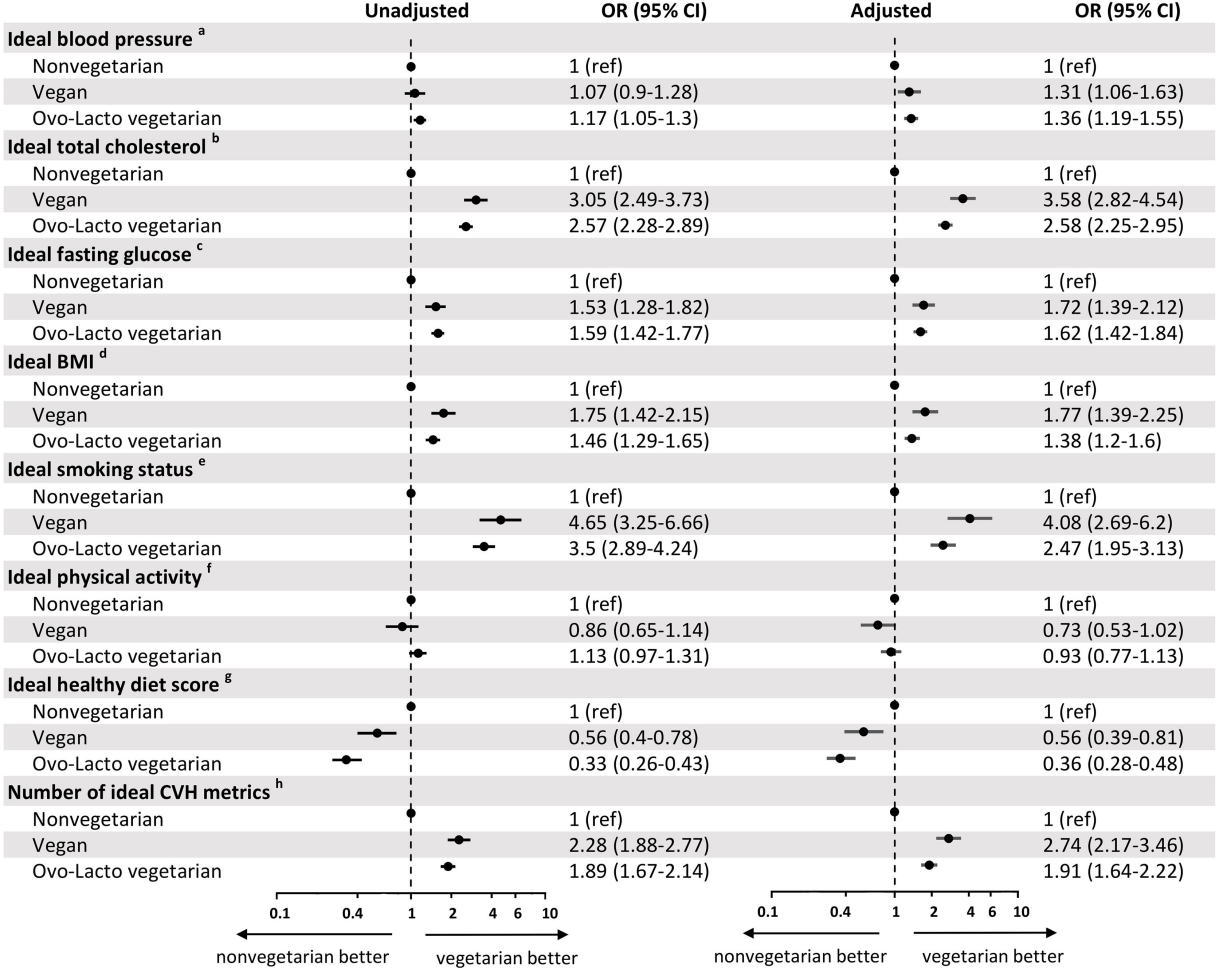
Odds ratio for cardiovascular health metrics associated with non-vegetarian, vegan, and ovo-lacto vegetarian populations. BP, blood pressure; SBP, systolic blood pressure; DBP, diastolic blood pressure; BMI, body mass index. ^a^Ideal blood pressure was defined as SBP < 120 mmHg and DBP < 80 mmHg. ^b^Ideal total cholesterol was defined as total cholesterol <200 mg/dL. ^c^Ideal fasting glucose was defined as fasting glucose <100 mg/dL. ^d^Ideal BMI was defined as BMI <25 kg/m^2^. ^e^Ideal smoking status was defined as never smokers. ^f^Ideal physical activity was defined as physical activity ≥210 min/week. ^g^Ideal healthy diet scores was defined as 4-5 components of healthy diet scores. ^h^Number of ideal CVH metrics was defined as 5–7 components of CVH metrics.

In Figure 3, we divided the vegetarian group into two subgroups, vegan and ovo-lacto-vegetarian groups, and showed the association with CVH metrics for each subgroup of vegetarians. In the unadjusted model, the vegan group showed better total cholesterol (OR = 3.05, 95% CI = 2.49–3.73), fasting glucose (OR = 1.53, 95% CI = 1.28–1.82), BMI (OR = 1.75, 95% CI = 1.42–2.15), and ideal cardiovascular health (OR = 2.28, 95% CI = 1.88–2.77) than the non-vegetarian group. A lower healthy diet score was found in the vegan group (OR = 0.56, 95% CI = 0.4–0.78). There was no difference in blood pressure (OR = 1.07, 95% CI = 0.9–1.28) or ideal physical activity (OR = 0.86, 95% CI = 0.65–1.14). After adjustment, these results were congruent, except that the vegan group tended to have a higher proportion of subjects with normal blood pressure (OR = 1.31, 95% CI = 1.06–1.63) than the non-vegetarian group.

Similarly, the ovo-lacto-vegetarian diet was related to an increased incidence of ideal vascular health (OR = 1.89, 95% CI = 1.67–2.14), normal blood pressure (OR = 1.17, 95% CI = 1.05–1.3), normal total cholesterol (OR = 2.57, 95% CI = 2.28–2.89), normal fasting glucose (OR = 1.59, 95% CI = 1.42–1.77), and normal BMI (OR = 1.46, 95% CI = 1.29–1.65), as well as a higher non-smoking ratio (OR = 3.5, 95% CI = 2.89–4.24) and lower healthy diet score (OR = 0.33, 95% CI = 0.26–0.43). Ideal physical activity did not differ significantly from that of non-vegetarians (OR = 1.13, 95% CI = 0.97–1.31). The analysis showed a similar trend after adjustment for confounders.

### Vegetarian Diet and Healthy Diet Score

Table 3 shows the association between vegetarian diets and different healthy dietary components. Due to the definition of vegetarianism, the fish intake was zero in the vegetarian group. For the other components, vegetarians were more likely to have a healthy dietary pattern, such as higher fruit and vegetable intake (OR = 2.27, 95% CI = 2.07–2.5), higher whole grain intake (OR = 2.6, 95% CI = 2.2–3.07), and lower sugar–sweetened beverage consumption (OR = 1.62, 95% CI = 1.43–1.84). There was no difference in sodium intake (OR = 0.79, 95% CI = 0.62–1). After multivariable adjustment, the analysis showed similar results. Vegetarians had higher fruit and vegetable intake (OR = 2.37, 95% CI = 2.12–2.64), higher whole grain intake (OR = 2.76, 95% CI = 2.28–3.35), and lower sugar–sweetened beverage consumption (OR = 1.36, 95% CI = 1.18–1.58), while there was no difference in sodium intake (OR = 0.86, 95% CI = 0.64–1.15).

**Table 3:** The odds ratio for healthy diet score between vegetarian and non-vegetarian.

	**Odds ratio (95% CI)^a^**	
	**Unadjusted**	**Adjusted^b^**
Fruits and vegetables (≥450 g/d)	2.27 (2.07–2.50)	2.37 (2.12–2.64)
Fish (≥198 g/week)^c^	–	–
Fiber–rich whole grains (≥85 g/d)	2.60 (2.20–3.07)	2.76 (2.28–3.35)
Sodium (<1,500 mg/d)	0.79 (0.62–1.00)	0.86 (0.64–1.15)
Sugar–sweetened beverages ( ≤ 1 liter/week)	1.62 (1.43–1.84)	1.36 (1.18–1.58)

a *Odds ratio and 95% CI were estimated by logistic regression models and using non-vegetarian as the reference group*.

b *Adjusted for age, gender, education level, and family income*.

c *The fish intake was zero in the vegetarian group*.

## Discussion

Our results demonstrate that vegetarian diets are associated with better CVH metrics than non-vegetarian diets. Ideal smoking status, ideal blood pressure, ideal total cholesterol, ideal serum glucose, and ideal BMI are more frequently observed among vegetarians, whereas there is no difference in physical activity. The study also revealed a favorable score on some separate healthy diet components, while the overall ideal healthy diet score was unfavorable in the vegetarian group due to the absence of fish intake. These findings can assist in guiding CVD prevention counseling for vegetarians.

The findings of our study corroborate the results of previous studies showing that vegetarian diets have beneficial effects on cardiometabolic risk factors, such as blood pressure, serum glucose, and lipids (9, 10, 31). Some plausible explanations for the benefit of vegetarian diets are considered. First, there are different nutrition profiles between vegetarian and non-vegetarian diets, and the nutrition profiles associated with vegetarian diets, such as higher levels of plant protein, fiber, and unsaturated fatty acids, may reduce cardiometabolic risks (32). A higher intake of fiber could reduce serum LDL cholesterol and prevent CVD by affecting the digestion and absorption of lipids in the intestine (33). Moreover, a lower content of saturated fatty acids in vegetarian diets affects the lipid profile by reducing LDL cholesterol and preventing atherosclerosis, which might contribute to lower blood pressure (34). Second, vegetarian diets are also rich in antioxidant agents that reduce oxidative stress and inflammation, thus promoting cardiovascular health (13). Third, vegetarians had a lower BMI in our study, and a lower BMI is beneficial in the context of cardiometabolic risk and fasting glucose control (35, 36). The functional adaptation of the cardiovascular system to increased metabolic demands and adipokine-related inflammation might be a possible mechanism for increased CVD risk in the higher BMI group (37). The high fiber content of the vegetarian diet contributed to a lower BMI because of delayed intestinal digestion and absorption (38). Therefore, the components of the vegetarian diet might lead to beneficial effects on blood pressure, serum glucose, and lipids. Together, these ideal CVH metrics could be instrumental in reducing the incidence of CVD.

In addition to cardiometabolic risk factors, our study assessed non-dietary habits, such as exercise and smoking, that influence cardiovascular risk and compared these factors between vegetarians and non-vegetarians. In the past, the effect of non-dietary habits was difficult to evaluate, and most studies did not report details on this issue; moreover, there is a potential bias when appraising the cardiovascular benefits between vegetarians and non-vegetarians if we did not consider non-dietary habits (39). In our study, physical activity was recorded based on the CVH metric, and less physical activity, with borderline significant differences, was detected in vegetarians after adjusting for potential covariates. This result indicated that the cardiovascular benefit of vegetarian diets might not be related to differences in the prevalence of physical activity between vegetarians and non-vegetarians. In contrast, the vegetarian group had a significantly lower proportion of smokers than the non-vegetarian group. This difference may be affected by religion or higher consciousness of health and lifestyle choices in the vegetarian group (40). Smoking is a potential confounder in the relationship between vegetarian diets and cardiovascular disease because tobacco use is also associated with an increased risk of CVD (41). The protective effect against CVD observed with vegetarian diets may be partially due to the lower smoking prevalence or even other healthy lifestyle choices and not only the vegetarian diet itself. Our study, which used a more comprehensive assessment than prior studies, provides stronger evidence to date that vegetarians had not only better metabolic profiles but also better lifestyle habits.

In our study, a vegetarian diet was found to have a beneficial effect on CVH metrics but a negative effect on healthy diet scores. Unsurprisingly, vegetarian individuals had difficulty meeting the ideal healthy diet metric because the importance of fish intake is highlighted in the CVH metrics. One of the components of the healthy diet score is a fish intake of ≥198 g/wk. Previous studies have suggested that omega-3 fatty acids in fish or fish oil supplements have protective effects and reduce the mortality rate of CVD (42–44). This is noteworthy from a clinical point of view because it is a potential disadvantage of vegetarian diets. Previous evidence showed that pesco-vegetarians (who consume fish and vegetables) had the lowest CVD mortality rate and that the CVD mortality rate was significantly different from that of other vegetarian subgroups (11). Even with the lower rate of the ideal healthy diet metric among vegetarians, the total beneficial effects on cardiometabolic risk factors were significantly higher and led to ideal CVH among vegetarians. A vegetarian diet can be a healthier dietary pattern than a non-vegetarian diet (13, 33), but it had a lower ideal CVD healthy diet score. This situation indicated two things. First, the healthy diet was a crucial but not exhaustive components of CVD prevention. Combined other healthy lifestyles or metabolic profiles were helpful for comprehensive assessment of CVH. Second, CVH metrics and healthy diet score should be used carefully when applied to vegetarians. Indeed, the CVH metric created by the AHA was designed to be used for general populations, not vegetarian populations. There are some limitations when assessing vegetarian by CVH metric. However, after comprehensive literature searches, we could not find another appropriate metric or score for vegetarian which evaluated cardiovascular health based on both metabolic risk factors and lifestyle behaviors. As the importance of a healthy diet and environmental impact is emphasized, an increasing number of people are starting to consume a plant-based diet (45). The CVH metric might need to be adjusted and place more emphasis on the importance of diet when applied to vegetarians. Fish consumption should be replaced by other omega-3 fatty acid-enriched foods, such as flaxseed and flaxseed oil, walnuts and walnut oil, and canola oil, when applied to vegetarians (46). To our knowledge, there is no evidence or study to support the adjustment of CVH metrics in vegetarian, and this is our future goal to develop a suitable CVH metric for vegetarian. From another point of view, adding a plant source or supplementation of omega-3 fatty acids to a vegetarian diet might be another suggestion for the purpose of achieving favorable cardiovascular health (46).

Subgroup analysis between ovo-lacto-vegetarian and vegan groups revealed consistent trends in the CVH metric. While Chiu et al. found different contents between vegan and ovo-lacto-vegetarian diets in a Taiwanese population (15), the benefit for cardiometabolic risk factors was still similar. We suggest that the different characteristics of vegetarian dietary patterns are not important factors influencing cardiometabolic factors. Our study was based on Taiwanese vegetarian diets, which are different from Western vegetarian diets. Unlike the Western vegetarian diet, which includes more beans, nuts, dairy products, eggs and lettuce, the Taiwanese vegetarian diet is mainly composed of rice, cooked vegetables, fruit, and soya products (14, 47). Despite the different components across cultures, the vegetarian diet showed a consistent protective effect against cardiometabolic risk. The common features of vegetarian diet, such as no red meat, lower saturated fatty acids, and higher fiber consumptions, may play an important role in cardiovascular health.

A significant strength of our study is that it involved a comprehensive assessment to prove that vegetarians had better metabolic profiles and lifestyle habits than non-vegetarians in Taiwan. However, there are some limitations that should be considered in the interpretation of the results. First, our analysis lacked a pesco-vegetarian group. Fish was one of the components in the healthy dietary score, and pesco-vegetarians showed the lowest CVD mortality among vegetarians in a previous study (11). However, due to the different dietary patterns, the population of pesco-vegetarians is small in Taiwan, and data are difficult to collect. The exact effect of fish on the vegetarian diet could not be evaluated in our study. Second, we did not take the reason for adoption of a vegetarian diet into consideration. As we mentioned above, some vegetarians in Taiwan adopt a vegetarian diet due to religion, rather than for health reasons (48). Some behaviors, such as not smoking, regular daily routines, and regular sleep schedules, that benefit CVD are promoted as religious disciplines. This factor may lead to potential bias in our analysis. Third, the duration of vegetarian diet consumption was not considered. The benefit on the CVH metric might be affected by the amount of time the vegetarian diet is followed. Fourth, the ideal BMI was defined as <25 kg/m^2^ in our study based on AHA guidelines, which was different from the recommendation of BMI between 18.5 and 24 kg/m^2^ from Taiwanese Health Promotion Administration. Using BMI cut-off point from AHA guidelines on Taiwanese population may be unsuitable and cause potential bias, especially when assessing under-weight population (BMI <18.5 kg/m^2^). Fifth, our study was cross-sectional. Thus, the temporal relationship was hard to explain. The changes in lifestyle and environment may be biased over a long sample collection period. A prospective, long-term, multinational study is needed to more precisely clarify the relationship between vegetarian diets and cardiovascular health metrics.

In conclusion, we found that vegetarian dietary patterns were significantly related to ideal CVH metrics and lower ideal healthy diet scores due to the lack of fish consumption. Vegetarian dietary patterns may have favorable effects on cardiovascular health. However, when assessing CVH metrics and healthy diet scores for vegetarians, metrics and scores chosen should be suitable for use with vegetarian populations.

## Data Availability Statement

The original contributions presented in the study are included in the article/Supplementary Material, further inquiries can be directed to the corresponding author/s.

## Ethics Statement

The studies involving human participants were reviewed and approved by Tri-Service General Hospital. The patients/participants provided their written informed consent to participate in this study. Written informed consent was obtained from the individual(s) for the publication of any potentially identifiable images or data included in this article.

## Author Contributions

Y-MH, W-LC, T-WK, and T-CP: study design. Y-MH, H-FY, W-LC, and T-CP: data collection. Y-MH, L-WW, H-FY, and T-CP: data analysis. Y-MH, W-LC, L-WW, and T-CP: drafting of the manuscript. All authors read and approved the final version of the manuscript.

## Funding

This study was supported by part of the National Defense Medical Center and Tri-Service General Hospital (TSGH-C107-164). The funders had no role in the study design, data collection and analysis, decision to publish, or preparation of the manuscript.

## Author Disclaimer

Any interpretation or conclusion described in this paper does not represent the views of the MJ Health Research Foundation.

## Conflict of Interest

The authors declare that the research was conducted in the absence of any commercial or financial relationships that could be construed as a potential conflict of interest.

## Publisher's Note

All claims expressed in this article are solely those of the authors and do not necessarily represent those of their affiliated organizations, or those of the publisher, the editors and the reviewers. Any product that may be evaluated in this article, or claim that may be made by its manufacturer, is not guaranteed or endorsed by the publisher.

## Abbreviations

CVD: cardiovascular disease
AHA: American Heart Association
CVH: cardiovascular health
LS7: Life's Simple 7
BMI: body mass index
BP: blood pressure
TC: total cholesterol
FG: fasting glucose
LDL: low-density lipoprotein.
