# Supplementary material for: Association Between Ideal Cardiovascular Health and Vegetarian Dietary Patterns Among Community-Dwelling Individuals
Source: Front Nutr. 2022 Mar 18;9:761982. doi: 10.3389/fnut.2022.761982 (PMC8971747; doi:10.3389/fnut.2022.761982)
Supplement: Supplementary file 1 [file Data_Sheet_1.docx]

**Supplemental table S1. Distribution of the participants by year**

| Year | n | Percent |
| --- | --- | --- |
| 2000 | 21 | 0.05 |
| 2001 | 990 | 2.14 |
| 2002 | 1310 | 2.83 |
| 2003 | 1513 | 3.27 |
| 2004 | 1680 | 3.63 |
| 2005 | 1919 | 4.15 |
| 2006 | 2290 | 4.95 |
| 2007 | 2406 | 5.20 |
| 2008 | 2933 | 6.34 |
| 2009 | 3761 | 8.13 |
| 2010 | 3543 | 7.65 |
| 2011 | 3571 | 7.71 |
| 2012 | 5418 | 11.71 |
| 2013 | 5620 | 12.14 |
| 2014 | 1809 | 3.91 |
| 2015 | 3223 | 6.96 |
| 2016 | 4280 | 9.25 |
| Total | 46287 | 100.00 |

**Supplemental table S2. Characteristics of nonvegetarian, vegan, and ovo-lacto vegetarian populations.**

| **Characteristics** | **All**  **(n =46287)** | **Vegan**  **(n=523)** | **Ovo-lacto Vegetarian**  **(n =1373)** | **Non-vegetarian**  **(n =44391)** | **P value** |
| --- | --- | --- | --- | --- | --- |
| **Continuous variables ^a^** |  |  |  |  |  |
| Age, years | 52.6 (10.1) | 56.3(10.8) | 56.1(10.8) | 52.5(10.0) | <0.01 |
| BMI, kg/m2 | 24.0 (3.4) | 23.0(3.3) | 23.2(3.3) | 24.0 (3.4) | <0.01 |
| Total cholesterol, mg/dL | 201.7 (35.6) | 178.8(34.6) | 182.7(33.0) | 202.5 (35.4) | <0.01 |
| Fasting sugar, mg/dL | 105.8 (24.3) | 103.7(25.4) | 102.6(20.8) | 106 (24.4) | <0.01 |
| Systolic pressure, mmHg | 121.4 (18.8) | 122.3(19.6) | 121.7(20.2) | 121.4 (18.7) | 0.46 |
| Diastolic pressure, mmHg | 74.5 (11.6) | 74.1(11.7) | 73.6(11.7) | 74.5 (11.5) | <0.01 |
| **Categorical variables ^b^** |  |  |  |  |  |
| Education | | | | | <0.01 |
| Below high school | 20866 (46.8) | 280 (56.3) | 749 (57.2) | 19837 (46.4) |  |
| beyond high school | 23681 (53.2) | 217 (43.7) | 560 (42.8) | 22904 (53.6) |  |
| Family income, NTD/year ^c^ | | | | | <0.01 |
| < 1.2 million | 24857 (68.2) | 335 (81.5) | 781 (75.0) | 23741 (67.9) |  |
| > 1.2 million | 11571 (31.8) | 76 (18.5) | 260 (25.0) | 11235 (32.1) |  |
| Sex | | | | | <0.01 |
| Male | 22987 (49.7) | 199 (38.0) | 440 (32.0) | 22348 (50.3) |  |
| Female | 23300 (50.3) | 324 (62.0) | 933 (68.0) | 22043 (49.7) |  |
| Blood pressure ^d^ | | | | | <0.01 |
| Poor | 8084 (17.5) | 99 (18.9) | 253 (18.4) | 7732 (17.4) |  |
| Intermedium | 17147 (37.0) | 179 (34.2) | 443 (32.3) | 16525 (37.2) |  |
| Ideal | 21056 (45.5) | 245 (46.8) | 677 (49.3) | 20134 (45.4) |  |
| Total cholesterol ^e^ | | | | | <0.01 |
| Poor | 6265 (13.5) | 30 (5.7) | 66 (4.8) | 6169 (13.9) |  |
| Intermedium | 16841 (36.4) | 104 (19.9) | 328 (23.9) | 16409 (37.0) |  |
| Ideal | 23181 (50.1) | 389 (74.4) | 979 (71.3) | 21813 (49.1) |  |
| Fasting glucose ^f^ | | | | | <0.01 |
| Poor | 3859 (8.3) | 39 (7.5) | 87 (6.3) | 3733 (8.4) |  |
| Intermedium | 21576 (46.6) | 197 (37.7) | 518 (37.7) | 20861 (47.0) |  |
| Ideal | 20852 (45.0) | 287 (54.9) | 768 (55.9) | 19797 (44.6) |  |
| Body mass index ^g^ | | | | | <0.01 |
| Poor | 2262 (4.9) | 18 (3.4) | 50 (3.6) | 2194 (5.0) |  |
| Intermedium | 13652 (29.5) | 106 (20.3) | 319 (23.2) | 13227 (29.8) |  |
| Ideal | 30373 (65.6) | 399 (76.3) | 1004 (73.1) | 28970 (65.3) |  |
| Smoking ^h^ | | | | | <0.01 |
| Poor | 7343 (15.9) | 14 (2.7) | 30 (2.2) | 7299 (16.4) |  |
| Intermedium | 3573 (7.7) | 21 (4.0) | 84 (6.1) | 3468 (7.8) |  |
| Ideal | 35371 (76.4) | 488 (93.3) | 1259 (91.7) | 33624 (75.7) |  |
| Physical activity ^i^ | | | | | 0.07 |
| Poor | 20940 (45.2) | 264 (50.5) | 623 (45.4) | 20053 (45.2) |  |
| Intermedium | 19283 (41.7) | 200 (38.2) | 552 (40.2) | 18531 (41.7) |  |
| Ideal | 6064 (13.1) | 59 (11.3) | 198 (14.4) | 5807 (13.1) |  |
| Healthy diet score ^j^ | | | | | <0.01 |
| Poor | 5416 (11.7) | 41 (7.8) | 112 (8.2) | 5263 (11.9) |  |
| Intermedium | 34905 (75.4) | 443 (84.7) | 1195 (87.0) | 33267 (74.9) |  |
| Ideal | 5966 (12.9) | 39 (7.5) | 66 (4.8) | 5861 (13.2) |  |
| Number of ideal CVH metrics | | | | | <0.01 |
| 0-2 | 15850 (34.2) | 99 (18.9) | 280 (20.4) | 15471 (34.9) |  |
| 3-4 | 23094 (49.9) | 273 (52.2) | 741 (54.0) | 22080 (49.7) |  |
| 5-7 | 7343 (15.9) | 151 (28.9) | 352 (25.6) | 6840 (15.4) |  |

^a^ Values in the continuous variables were expressed as mean (standard deviation) and analyzed by Student’s t test.

^b^ Values in the categorical variables were expressed as number (%) and analyzed by the chi-square test.

^c^ Values were expressed as New Taiwan dollar (NTD)

^d^ Ideal, intermediate, poor BP were defined as SBP <120 mmHg and DBP <80 mmHg, SBP 120-139 mmHg or DBP 80-89 mmHg or treated to goal, and SBP ≥140 mmHg or DBP ≥90 mmHg, respectively.

^e^ Ideal, intermediate, poor total cholesterol were defined as <200 mg/dL, 200-239 mg/dL or treated to goal, and 240 mg/dL, respectively.

^f^ Ideal, intermediate, poor fasting glucose were defined as <100 mg/dL, 100-125 mg/dL, and≥126 mg/dL, respectively.

^g^ Ideal, intermediate, poor BMI were defined as <25 kg/m^2^, 25-29.9 kg/m^2^, and ≥30 kg/m^2^, respectively.

^h^ Ideal, intermediate, poor smoking status were defined as never smokers, past smokers, and current smokers, respectively.

^i^ Ideal, intermediate, and poor physical activity were defined as ≥ 210 min/week, 60-210 min/week, and < 60 min/week, respectively.

^j^ Ideal, intermediate, and poor healthy diet scores were defined as 4-5 components, 2-3 components, and 0-1 components, respectively.

**Supplemental table S3. STROBE-nut: An extension of the STROBE statement for nutritional epidemiology**

Lachat C et al. (2016) STrengthening the Reporting of OBservational studies in Epidemiology – Nutritional Epidemiology (STROBE-nut): an extension of the STROBE statement. Plos Medicine 13(6) <http://dx.doi.org/10.1371/journal.pmed.1002036> [pdf](http://journals.plos.org/plosmedicine/article/asset?id=10.1371%2Fjournal.pmed.1002036.PDF) or [online](http://journals.plos.org/plosmedicine/article?id=10.1371/journal.pmed.1002036) version.

| **Item** | **Item nr** | **STROBE recommendations** | **Extension for Nutritional Epidemiology studies (STROBE-nut)** | **Reported on page #** |
| --- | --- | --- | --- | --- |
| **Title and**  **abstract** | 1 | (a) Indicate the study’s design with a commonly used term in the title or the abstract.  (b) Provide in the abstract an informative and balanced summary of what was done and what was found. | **nut-1** State the dietary/nutritional assessment method(s) used in the title, abstract, or keywords. | Abstract p. 3-4 |
| **Introduction** |  |  |  |  |
| Background rationale | 2 | Explain the scientific background and rationale for the investigation being reported. |  | Introduction p. 5 |
| Objectives | 3 | State specific objectives, including any pre-specified hypotheses. |  | Introduction p.5-6 |
| **Methods** |  |  |  |  |
| Study design | 4 | Present key elements of study design early in the paper. |  | Methods (study population and design) p.7 |
| Settings | 5 | Describe the setting, locations, and relevant dates, including periods of recruitment, exposure, follow-up, and data collection. | **nut-5** Describe any characteristics of the study settings that might affect the dietary intake or nutritional status of the participants, if applicable. | Methods (study population and design) p.7 |
| Participants | 6 | a) Cohort study—Give the eligibility criteria, and the sources and methods of selection of participants. Describe methods of follow-up.  Case-control study—Give the eligibility criteria, and the sources and methods of case ascertainment and control selection. Give the rationale for the choice of cases and controls.  Cross-sectional study—Give the eligibility criteria, and the sources and methods of selection of participants.  (b) Cohort study—For matched studies, give matching criteria and number of exposed and unexposed.  Case-control study—For matched studies, give matching criteria and the number of controls per case. | **nut-6** Report particular dietary, physiological or nutritional characteristics that were considered when selecting the target population. | Methods (study population and design) p.7 |
| Variables | 7 | Clearly define all outcomes, exposures, predictors, potential confounders, and effect modifiers. Give diagnostic criteria, if applicable. | **nut-7.1** Clearly define foods, food groups, nutrients, or other food components.  **nut-7.2** When using dietary patterns or indices, describe the methods to obtain them and their nutritional properties. | Methods (sociodemographic data, dietary data, cardiovascular health metrics) p.8-10 |
| Data sources - measurements | 8 | For each variable of interest, give sources of data and details of methods of assessment (measurement).Describe comparability of assessment methods if there is more than one group. | **nut-8.1** Describe the dietary assessment method(s), e.g., portion size estimation, number of days and items recorded, how it was developed and administered, and how quality was assured. Report if and how supplement intake was assessed.  **nut-8.2** Describe and justify food composition data used. Explain the procedure to match food composition with consumption data. Describe the use of conversion factors, if applicable.  **nut-8.3** Describe the nutrient requirements, recommendations, or dietary guidelines and the evaluation approach used to compare intake with the dietary reference values, if applicable.  **nut-8.4** When using nutritional biomarkers, additionally use the STROBE Extension for Molecular Epidemiology (STROBE-ME). Report the type of biomarkers used and their usefulness as dietary exposure markers.  **nut-8.5** Describe the assessment of nondietary data (e.g., nutritional status and influencing factors) and timing of the assessment of these variables in relation to dietary assessment.  **nut-8.6** Report on the validity of the dietary or nutritional assessment methods and any internal or external validation used in the study, if applicable. | Methods (dietary data, cardiovascular health metrics) p.8-10 |
| Bias | 9 | Describe any efforts to address potential sources of bias. | **nut-9** Report how bias in dietary or nutritional assessment was addressed, e.g., misreporting, changes in habits as a result of being measured, or data imputation from other sources | Not applicable |
| Study Size | 10 | Explain how the study size was arrived at. |  | Methods (study population and design) p.7 |
| Quantitative variables | 11 | Explain how quantitative variables were handled in the analyses. If applicable, describe which groupings were chosen and why. | **nut-11** Explain categorization of dietary/nutritional data (e.g., use of N-tiles and handling of nonconsumers) and the choice of reference category, if applicable. | Methods (dietary data, statistical analysis) p.8-10 |
| Statistical  Methods | 12 | (a) Describe all statistical methods, including those used to control for confounding  (b) Describe any methods used to examine subgroups and interactions.  (c) Explain how missing data were addressed.  (d) Cohort study—If applicable, explain how loss to follow-up was addressed.  Case-control study—If applicable, explain how matching of cases and controls was addressed.  Cross-sectional study—If applicable, describe analytical methods taking account of sampling strategy.  (e) Describe any sensitivity analyses. | **nut-12.1** Describe any statistical method used to combine dietary or nutritional data, if applicable.  **nut-12.2** Describe and justify the method for energy adjustments, intake modeling, and use of weighting factors, if applicable.  **nut-12.3** Report any adjustments for measurement error, i.e,. from a validity or calibration study. | Methods (statistical analysis) p.10 |
| **Results** |  |  |  |  |
| Participants | 13 | (a) Report the numbers of individuals at each stage of the study—e.g., numbers potentially eligible, examined for eligibility, confirmed eligible, included in the study, completing follow-up, and analyzed.  (b) Give reasons for non-participation at each stage.  (c) Consider use of a flow diagram. | **nut-13** Report the number of individuals excluded based on missing, incomplete or implausible dietary/nutritional data. | Methods (study population and design) p. 7  Figure 1  Result (demographic) p.11 |
| Descriptive data | 14 | (a) Give characteristics of study participants (e.g., demographic, clinical, social) and information on exposures and potential confounders  (b) Indicate the number of participants with missing data for each variable of interest  (c) Cohort study—Summarize follow-up time (e.g., average and total amount) | **nut-14** Give the distribution of participant characteristics across the exposure variables if applicable. Specify if food consumption of total population or consumers only were used to obtain results. | Result (demographic) p.11  Table 1 |
| Outcome data | 15 | Cohort study—Report numbers of outcome events or summary measures over time.  Case-control study—Report numbers in each exposure category, or summary measures of exposure.  Cross-sectional study—Report numbers of outcome events or summary measures. |  | Result (demographic) p.11  Table 1, Figure 2 |
| Main results | 16 | (a) Give unadjusted estimates and, if applicable, confounder-adjusted estimates and their precision (e.g., 95% confidence interval).  Make clear which confounders were adjusted for and why they were included.  (b) Report category boundaries when continuous variables were categorized.  (c) If relevant, consider translating estimates of relative risk into absolute risk for a meaningful time period. | **nut-16** Specify if nutrient intakes are reported with or without inclusion of dietary supplement intake, if applicable. | Result (Vegetarian diet and cardiovascular health metric) p.11-12  Table 2 |
| Other analyses | 17 | Report other analyses done—e.g., analyses of subgroups and interactions and sensitivity analyses. | **nut-17** Report any sensitivity analysis (e.g., exclusion of misreporters or outliers) and data imputation, if applicable. | Result (Vegetarian diet and cardiovascular health metric, Vegetarian diet and healthy diet score) p.11-13  Table 3, Figure 3 |
| **Discussion** |  |  |  |  |
| Key results | 18 | Summarize key results with reference to study objectives. |  | Discussion (1st paragraph) p.14 |
| Limitation | 19 | Discuss limitations of the study, taking into account sources of potential bias or imprecision. Discuss both direction and magnitude of any potential bias. | **nut-19** Describe the main limitations of the data sources and assessment methods used and implications for the interpretation of the findings. | Discussion (6^th^ paragraph) p.16-17 |
| Interpretation | 20 | Give a cautious overall interpretation of results considering objectives, limitations, multiplicity of analyses, results from similar studies, and other relevant evidence. | **nut-20** Report the nutritional relevance of the findings, given the complexity of diet or nutrition as an exposure. | Discussion p.14-17 |
| Generalizability | 21 | Discuss the generalizability (external validity) of the study results. |  | Discussion (7^th^ paragraph) p.17 |
| **Other information** |  |  |  |  |
| Funding | 22 | Give the source of funding and the role of the funders for the present study and, if applicable, for the original study on which the present article is based. |  | Financial support p.2 |
| *Ethics* |  |  | **nut-22.1** Describe the procedure for consent and study approval from ethics committee(s). | Methods (Study population and design) p.7 |
| *Supplementary material* |  |  | **nut-22.2** Provide data collection tools and data as online material or explain how they can be accessed. | Methods (Study population and design) p.7  Supplemental table S2 |
